# A rare case of extrauterine schwannoma-like leiomyoma after transvaginal hysterectomy: laparoscopic management

**DOI:** 10.52054/FVVO.14.3.039

**Published:** 2022-09-30

**Authors:** M Medvediev, A Tinelli

**Affiliations:** Dnipro State Medical University, Dnipro, Ukraine, 49044; Department of Obstetrics and Gynecology and CE- RICSAL (CEntro di RIcerca Clinico SALentino), “Veris delli Ponti” Hospital, Scorrano, Lecce, Italy, e-mail: andreatinelli@gmail.com

**Keywords:** Schwannoma, leiomyoma, laparoscopy, diagnosis

## Abstract

**Background:**

Schwannomas (neurilemomas) are encapsulated tumours made entirely of benign neoplastic Schwann cells. They are the most common tumour of peripheral nerves, but very uncommon in gynaecologic practice.

**Objectives:**

The objective is to demonstrate unusual histology mimicking schwannoma in a case of leiomyoma in a woman who had a history of vaginal hysterectomy.

**Materials and Methods:**

We report a case of a 50-year-old hysterectomised patient who was referred with complaints of dull pain in the left inguinal region of the abdominal cavity during the last 3 months. The narrated surgical video article demonstrates the dissection of the left parametrium, tumour removal, colpotomy, specimen extraction, and vaginal laparoscopic suturing. Pre-operative CT-scan images of the pelvis with retroperitoneal tumour and macroscopic and histological views of the schwannoma are also provided.

**Main outcome measures:**

Full recovery of the patient after laparoscopic removal of the tumour.

**Results:**

As a result of the surgical treatment, the patient recovered fully.

**Conclusions:**

Schwannoma-like leiomyomas are rare tumours. They can be extra peritoneally located, without any connection to the uterus and adnexa. They are difficult to diagnose before surgery. The laparoscopic approach is the best option for the treatment of such rare extra organic tumours.

## Introduction

Schwannomas (neurilemomas) are encapsulated tumours made entirely of benign neoplastic Schwann cells. They are the most common tumour of peripheral nerves. Schwannoma-like leiomyoma located in the pelvic area is an extremely rare benign tumour (Morales et al., 2017; Lee et al., 2002). Since these tumours are very rare, pre-operative diagnosis is difficult, and their characteristics are often only determined after surgery and histological examination. Laparoscopy is a method of choice even in cases where there is no certainty about the histological nature of the tumour, as it allows one to perform the procedure precisely and extract the specimen using safe techniques. In this video article, we describe our case of laparoscopic management of retroperitoneal pelvic schwannoma in a woman who had previously had a vaginal hysterectomy for the leiomyomatous uterus 3 years earlier. A comparison of radiological data with a macroscopic view of the tumour presented in the video could help readers to suspect such a diagnosis in the future. Colpotomy as an alternative to power morcellation is discussed.

## Learning objective

To get acquainted with a successful case of the surgical treatment of a retroperitoneal tumour of unknown structure confirmed as a schwannoma after histological evaluation.

## Patients and methods

We present a case of a 50-year-old patient with this pathology, who was referred with complaints of dull pain in the left inguinal region of the abdominal cavity during the last 3 months.

The transvaginal hysterectomy with bilateral salpingectomy was performed 3 years before clinical presentation due to symptomatic uterine leiomyoma. The surgical procedure was typical and without any findings suggesting the presence of an extrauterine tumour. The initial surgery was performed by the same surgeon (MM). The post-operative course was uneventful, and the patient didn’t attend a gynaecologist.

The initial radiological diagnosis was “left ovarian mass”, which was excluded thereafter using a contrast-enhanced pelvic CT scan. Differential diagnosis included: ovarian tumour; myoma of the round ligament or parasitic myoma; foreign body after previous surgery; extraperitoneal tumour of unknown origin.

The decision about surgery was discussed with the patient. There were two indications: pain and tumour of unknown origin with the possibility of malignancy.

After the surgery, the histologic evaluation using standard staining with haematoxylin-eosin with magnification X 100 was performed.

## Results

For better visualisation and the possibility to examine the whole abdominal area, a laparoscopic approach was utilised. Then step-by-step the left paravesical space was developed and tumour removal was performed using the classical laparoscopic technique with bipolar grasper and scissors. We used conventional instruments including bipolar forceps, scissors, and grasping forceps. We used three 5mm working trocars and one 10mm optic trocar to optimise surgery ergonomics. Three working instruments were used to help to improve dissection precision, especially in cases of unknown extraperitoneal tumours.

There was no significant bleeding. The tumour was not densely adherent to any structure in the pelvic cavity, and it was quite easy to remove it without it fragmenting.

The tumour was removed through a colpotomy incision. At the end of the procedure, the colpotomy was sutured laparoscopically using a purse-string suture. Polyglactin 910 2-0 thread on CT-1 needle was utilised (see video).

The surgical time was 37 mins. Intraoperative blood loss was 25 ml. No early or late postoperative complications were observed. The patient didn’t receive any blood products or postoperative treatment except for nonsteroidal anti-inflammatory drugs. The patient was discharged 24 hours after the surgery. Full recovery was achieved in 7 days after the surgery.

The tumour size was 8 x 4 x 4.5cm cm with a clear capsule. It was rather cystic with thick dense walls approximately 1 cm and grey in colour. The central portion was solid, cartilage-like and yellowish in colour.

The histology showed the tumour was built from multidirectional bundles of spindle-shaped cells. Tumour cells had fuzzy borders, with a moderate volume of eosinophilic cytoplasm, ovoid, and elongated nuclei. Additionally, the immunohistochemical staining for desmin and h-caldesmon was implemented with a positive cytoplasmic reaction in leiomyoma cells (see video). The patient has been observed for 1 year after surgery. There were no significant complaints and no relapse on sonographic check-up a year after the surgery.

## Discussion

The neurilemmoma-like or schwannoma-like leiomyomas of the uterine body are very rare, they are mostly diagnosed in the gastrointestinal tract. Even though it was necessary to conduct a differential diagnosis considering many variants of uterine leiomyoma, including a parasitic tumour, histological examination made it possible to clarify the diagnosis (López-Ruiz et al., 2018; Tahmasbi et al., 2012; Manjula et al., 2011).

Power morcellation has undergone scrutiny recently because of concerns that it can disseminate occult uterine sarcoma, other undiagnosed malignancies, and benign tissue. To limit uterine tissue dissemination, morcellation may be contained within a bag (Odejinmi et al., 2019).

Bag extraction is prudent in all cases when extraction of the whole specimen is impossible and intraabdominal morcellation is needed or in cases of the specimen’s friable structure. In our case, it was decided to remove the tumour through a colpotomy incision avoiding risks of morcellation and without the bag because of the dense elastic structure of the specimen without any clinical suspicion of malignancy and because of the size, which corresponded to the size of colpotomy incision.

The risk of recurrence in this case and this type of tumour is very low, however regular annual gynaecologic check-ups are advised.

## Conclusions

In conclusion, Schwannoma-like leiomyoma can be extra peritoneally located, without any connection to the uterus and adnexa. This rare tumour is difficult to diagnose before surgery. The laparoscopic approach is the best option for the treatment of such rare extra organic tumours.

## Video scan (read QR)

https://vimeo.com/749074872/a50f9373e2

**Figure:**
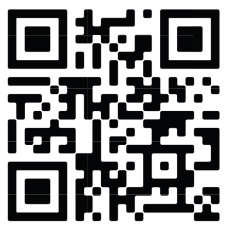
QR code resolving to https://vimeo.com/749074872/a50f9373e2
